# Modulation Recognition of Radar Signals Based on Adaptive Singular Value Reconstruction and Deep Residual Learning

**DOI:** 10.3390/s21020449

**Published:** 2021-01-10

**Authors:** Kuiyu Chen, Shuning Zhang, Lingzhi Zhu, Si Chen, Huichang Zhao

**Affiliations:** School of Electronic and Optical Engineering, Nanjing University of Science and Technology, Nanjing 210094, China; cky@njust.edu.cn (K.C.); zhulingzhi@njust.edu.cn (L.Z.); chensi354@njust.edu.cn (S.C.); zhaohch@njust.edu.cn (H.Z.)

**Keywords:** radar signals, modulation recognition, adaptive singular value reconstruction, deep residual learning

## Abstract

Automatically recognizing the modulation of radar signals is a necessary survival technique in electronic intelligence systems. In order to avoid the complex process of the feature extracting and realize the intelligent modulation recognition of various radar signals under low signal-to-noise ratios (SNRs), this paper proposes a method based on intrapulse signatures of radar signals using adaptive singular value reconstruction (ASVR) and deep residual learning. Firstly, the time-frequency spectrums of radar signals under low SNRs are improved after ASVR denoising processing. Secondly, a series of image processing techniques, including binarizing and morphologic filtering, are applied to suppress the background noise in the time-frequency distribution images (TFDIs). Thirdly, the training process of the residual network is achieved using TFDIs, and classification under various conditions is realized using the new-trained network. Simulation results show that, for eight kinds of modulation signals, the proposed approach still achieves an overall probability of successful recognition of 94.1% when the SNR is only −8 dB. Outstanding performance proves the superiority and robustness of the proposed method.

## 1. Introduction

Nowadays, advanced electronic reconnaissance technology is the key to obtain superiority in electronic countermeasures [1,2]. Meanwhile, intrapulse modulation recognition of radar signals is one of the crucial components in electronic reconnaissance. Higher-precision recognition of radar signal modulation types means more effectiveness to judge the threat level of received signals and better accuracy to estimate parameters of the detected signals. Therefore, the study of the intrapulse modulation recognition of radar signals is of great significance. However, the working conditions of modern radars contain a large amount of noise, which makes the identification of radar signals more and more difficult. Hence, the method for the intrapulse modulation recognition of radar signals needs to have a better performance under low signal-to-noise ratios (SNRs) [3].

Researchers have been exploring methods for the intrapulse modulation recognition of radar signals for many years. Current studies mainly focus on recognition methods based on statistical patterns consisting of two key steps: feature extraction and classification. Signal characteristics are extracted based on the frequency spectrum [4], high-order cumulant [5] and so on for classification.

In [6], box dimension and information dimension, which characterize the complexity of the signal, have been extracted in the bispectrum of signals as recognition features. However, this method can hardly recognize radar signals effectively under low SNRs. Literature [7] has extracted the ratio of the minimum to the maximum of Hough transform results as well as the peak number of the Hough transform of the real part of the Rihaczek distribution as the input features of the classifier. Although this recognition system has a better antinoise performance, the system only considers the classification of limited modulations. In [8,9], the high-order cumulant, the instantaneous frequency and all order moments were extracted in the time-frequency domain. Based on these features, the system can accurately identify eight kinds of radar signals in an environment full of intense noise. However, these methods all need researchers to extract features that can reflect the difference between several types of radar signals in advance. Hence, the discrimination of features, which is highly dependent on the experience of researchers, determines the final classification accuracy. To get rid of the dependence on the experience of researchers, a method that can identify radar signals without extracting features is urgent to be proposed.

With the rapid development of artificial intelligence, deep learning has contributed to significant breakthroughs in speech recognition, natural language processing and image recognition [10,11,12,13,14]. This is mainly due to its ability to automatically learning features from the input. Hence, it has great significance to apply deep learning to recognizing radar signals. As one of the most successful developments from deep learning, a deep convolutional neural network (DCNN) can learn features from inputs automatically. This avoids the complex process of feature extraction. Compared with the traditional methods, the problem of searching for appropriate features is transformed into the issue of image classification by DCNN. Concerning modulation recognition of signals based on DCNN, some studies have been done.

In [15], a DCNN based on samples composed of in-phase and quadrature component signals has been designed to identify modulation signals, which are relatively easy to identify. Another DCNN has been trained on constellation diagrams, which can identify 16 quadratic-amplitude modulation (QAM) and 64 QAM signals. Literature [16] used a low-rank representation of the cyclic spectra of modulated signals as the input of DCNN to recognize various signals. This approach can recognize binary phase-shift keying (BPSK), quadrature phase-shift keying (QPSK), frequency-shift keying (FSK), 4FSK, minimum shift keying (MSK), amplitude modulation (AM) and frequency modulation (FM) signals. The overall correct recognition rate is over 95% when the SNR is above 2 dB. Literature [17] transformed raw signal sequences in the autocorrelation domain, and then designed a DCNN to train autocorrelation sequences. This classification system can classify FSK, BPSK, continuous wave (CW), linear frequency modulation (LFM), Sinusoidal frequency modulation (SFM) and QPSK signals. The accuracy of all six kinds of signals is close to 100% when the SNR is above −2 dB. To identify BPSK, LFM, Costas, Frank code and T1-T4 signals, literature [18] introduced the time-frequency distribution images (TFDIs) of radar signals to LeNet-5. The total probability of successful recognition of this automatic waveform recognition system can exceed 93% at the SNR of −2 dB. It can be seen that the TFDIs-based DCNN has the most satisfactory recognition performance. However, when the SNR is lower, the recognition accuracy is unable to be guaranteed. This is mainly caused by the large amount of noise which will have a bad effect on the feature-learning process of DCNN. Thus, the denoising preprocessing and a network that can capture features more precisely are crucial to the final recognition accuracy.

To get out of the predicament of inability to recognize radar signals under low SNRs, an adaptive singular value reconstruction (ASVR) algorithm based on the singular value difference spectrum is proposed for the first time in this paper. Singular value reconstruction is a reconstruction algorithm based on singular value decomposition (SVD). It selects singular values and corresponding singular vectors which represent the useful signal components to reconstruct signal [19]. Most noises are filtered out after reconstruction. Due to its excellent noise suppression capability, SVR is widely used in various fields, such as image processing, data reduction and signal denoising [20,21,22]. However, in the background of intense noise, these methods cannot find the correct number of useful singular values. Thus, these approaches can hardly eliminate the noise under low SNRs. In view of this problem, based on the demarcation point in the singular value difference spectrum, the ASVR algorithm is designed to select the number of useful singular values adaptively to repair signals under low SNRs in this paper. The TFDI quality of the restored signal though ASVR is significantly improved, which makes it easy to identify radar signals under low SNRs.

Besides, one of the most successful structures of DCNNs called residual learning networks is applied in this paper to strengthen the network learning ability. Combining ASVR and the deep residual learning, accurate recognition of eight types of radar signals (LFM, SFM, even quadratic frequency modulation (EQFM), FSK, 4FSK, BPSK, Frank code and CW signals) is realized in four main steps. Firstly, received raw signals are reconstructed by ASVR to remove the noise in signals. Secondly, smooth pseudo-Wigner-Ville distribution (SPWVD) transformation is utilized to obtain the TFDIs of repaired signals. Thirdly, image processing methods, including binarizing and morphologic filtering, are applied to remove the background noise of the TFDIs. Finally, after finishing the off-line training of the deep residual network using TFDIs, recognition of eight types of radar signals is achieved with high accuracy. The simulation results prove the superiority and robustness of the method in this paper.

This paper is organized as follows. Section 2 introduces the radar signal model and the recognizing system overview. The data processing method is proposed in Section 3. Section 4 presents the deep residual network in detail. Section 5 analyzes the classification results under different conditions. The comparisons between the proposed approach and the previous method are also given in this section. Finally, Section 6 summarizes the whole paper.

## 2. Signal Model and System Overview

### 2.1. Signal Model

**Figure 1:**
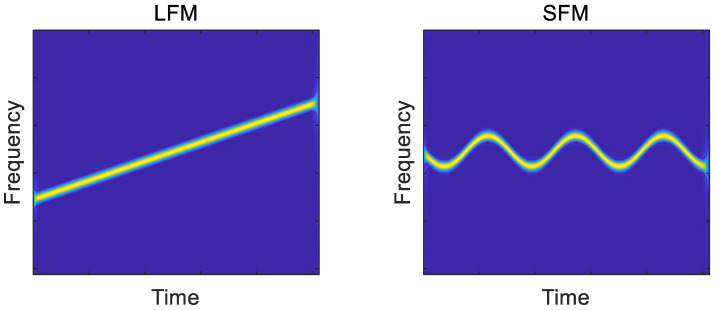

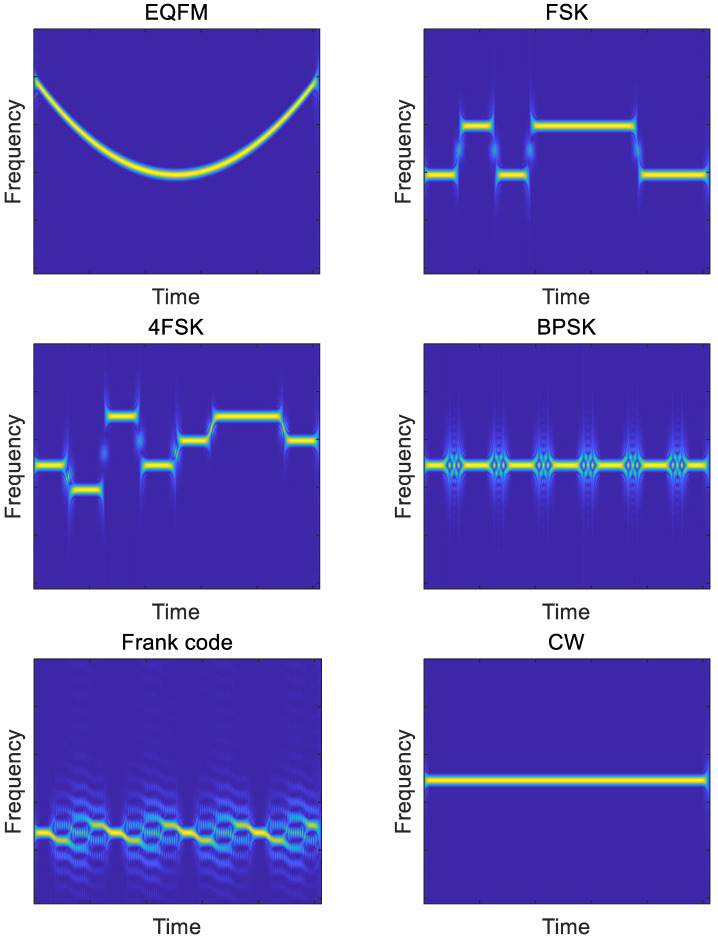
The TFDIs of 8 typical radar signals.

The received radar signal is disturbed by additive noise. Its model can be expressed as below

(1) $$y(t)=s(t)+n(t)=A\cdot \mathrm{rect}\left(t/T\right)e^{j\left(2\pi f_{0}t+\phi_{t}+\phi_{0}\right)}+n(t)$$

where y(t) is the received signal, s(t) is the modulated signal and n(t) is the channel noise. The channel noise is generally assumed to be additive white Gaussian noise with the variance $\sigma_{\varepsilon }^{2}$. A, T, $f_{0}$, $\phi_{0}$ represent the amplitude, the pulse width, the carrier frequency and the initial phase, respectively. $\phi_{t}$ is the phase modulation function, which has a significant difference among various radar signals. In this study, radar signals are classified into four classes: frequency modulation signals (LFM, SFM, EQFM), discrete frequency codes signals (FSK, 4FSK), phase codes signals (BPSK, Frank code) and continuous wave signal (CW). In Figure 1, the TFDIs of radar signals are presented.

### 2.2. System Overview 

**Figure 2:**
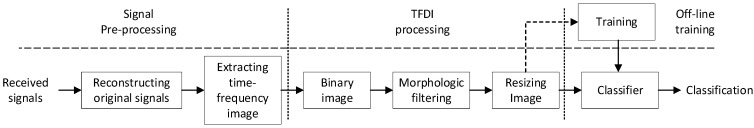
The structure diagram of the recognition system.

As is shown in Figure 2, this paper designs a radar signal intrapulse modulation recognition system. The system consists of three parts, including signal preprocessing, TFDIs processing and the classifier. 

In the part of signal preprocessing, received signals are reconstructed by the ASVR algorithm. The noise has been removed to a great degree in reconstructed signals, while the characteristics of instantaneous frequency in original signals are retained. Therefore, the quality of the TFDIs that are extracted via SPWVD transformation is much improved.

However, the obtained TFDIs still contain some background noise under low SNRs, which will contribute to the decrease in identification accuracy. Thus, image processing algorithms are used to smooth TFIDs and reduce noise. Firstly, the TFDIs are transformed into binary images based on the Otsu method. Then, morphologic filters are applied to smooth the main body boundary and eliminate small objects in binary images. After ensuring the quality of images, the system resizes the image to an appropriate size by utilizing bicubic interpolation.

Finally, the system uses the denoised TFDIs as the input of the deep residual network and trains the network off-line. After realizing the residual network training, the classification of various modulations of radar signals can be achieved with high accuracy.

## 3. Data Processing 

### 3.1. Signal Preprocessing

The TFDIs of the radar signals, obtained under low SNRs, contains a large amount of noise so that the characteristics of the TFDI are drowned. Thus, the classifier can hardly recognize the TFDIs accurately. In order to improve the recognition accuracy, preserving the features of TFDIs while suppressing the noise is of great significance. This paper proposes an ASVR algorithm to eliminate the noise and improve the time-frequency spectrums.

#### 3.1.1. Singular Value Decomposition

Assuming that $y=\{y_{1},y_{2},\cdots ,y_{N}\}$ is a discrete one-dimensional time sequence, M×K Hankel matrix of y [23] is determined as below

(2) $$H=\left[\begin{array}{cccc} y_{1} & y_{2} & \cdots & y_{M}\\ y_{2} & y_{3} & \cdots & y_{M+1}\\ \vdots & \vdots & \ddots & \vdots \\ y_{K} & y_{K+1} & \cdots & y_{K+M-1} \end{array}\right]$$

where K∈[1,N/2], N=K+M−1. The SVD [24] of the matrix H can be performed according to (3).

(3) $$H=U\Sigma V^{T}$$

where U and V are the singular vectors matrixes of H($UU^{H}=I$, $VV^{H}=I$). Only the diagonal elements of Σ have non-negative and real values. These values are also called singular values. Corresponding to the proportion of the signal component energy, singular values can be expressed as follows

(4) $$\delta_{i}=\left[\delta_{1},\delta_{2},\cdots ,\delta_{M}\right];\;\;\delta_{1}\geq \delta_{2}\geq \cdots \geq \delta_{M}$$

#### 3.1.2. Matrix Perturbation Theory

According to (1), the one-dimensional time sequence $Y_{N}=S_{N}+n_{N}$, where $S_{N}$ is the modulated signal sequence, $n_{N}$ is white Gaussian noise sequence and N is the length of the sequence. Assuming that positive integer K is the length of the sliding window where 1<K<N, the track matrix X of the sequence $Y_{N}$ is obtained by embedding operation.

(5) $$X=\left[X_{1},X_{2},\cdots ,X_{M}\right]\in R_{K\times M}$$

where M=N−K+1. Let X=S+E. S is the track matrix of the useful signal and E is the track matrix of the noise, that is

(6) $$XX^{T}=\left(S+E\right){\left(S+E\right)}^{T}$$

Because Gaussian noise has the same variance $\sigma_{\varepsilon }^{2}$ and there is no statistical correlation between Gaussian noise and useful signal, that is

(7) $$XX^{T}=SS^{T}+\sigma_{\varepsilon }{}^{2}I$$

where I is the identity matrix. Let $\mathrm{rank}(SS^{T})=r\leq M$, if eigenvalue decomposition of $SS^{T}$ is $SS^{T}=U\Lambda U^{T}$, where U is the matrix of eigenvectors. The eigenvalue decomposition of $XX^{T}$ can be expressed as

(8) $$XX^{T}=U\Lambda U^{T}+\sigma_{\varepsilon }{}^{2}I=U(\Lambda +\sigma_{\varepsilon }{}^{2}I)U^{T}=U\Sigma U^{T}$$

where $\Lambda =\mathrm{diag}[\lambda_{1}{}^{2}\;\lambda_{2}{}^{2}\;\cdots \;\lambda_{r}{}^{2}\;0\;\cdots \;0]$, and $\lambda_{1}{}^{2}\geq \lambda_{2}{}^{2}\geq \cdots \lambda_{r}{}^{2}$ are nonzero eigenvalues of $SS^{T}$. Hence, Σ can be expressed as follows

(9) $$\Sigma =\Lambda +\sigma_{\varepsilon }{}^{2}I=\mathrm{diag}[\lambda_{1}{}^{2}+\sigma_{\varepsilon }{}^{2}\;\lambda_{2}{}^{2}+\sigma_{\varepsilon }{}^{2}\;\cdots \;\lambda_{r}{}^{2}+\sigma_{\varepsilon }{}^{2}\;\sigma_{\varepsilon }{}^{2}\;\cdots \;\sigma_{\varepsilon }{}^{2}]$$

It can be inferred from (9) that a few r(r<M) larger singular values at first represent the useful signal components with primary energy. The later M−r smaller singular values represent noise components. In this way, the signal is grouped to realize the signal-noise separation by choosing the appropriate number of r [25]. 

An appropriate algorithm to objectively select the number of useful singular values is vital to reconstructing the signal. Literature [19] puts forward the singular value average method to obtain the number of useful singular values. The singular values which are larger than the average value are chosen as the useful ones, but the method is effective only when the original signal is polluted by the slight noise. Literature [26] determines the number of useful singular values by the number of primary frequencies in the results of the fast Fourier form (FFT). This method is only effective for the signals with evident primary frequency components. For the frequency modulated signals, the number of the main frequencies cannot be determined correctly by the results of FFT. Noise also makes FFT results impossible to be extracted. Especially in the background with intense noise, these methods will fail.

#### 3.1.3. Adaptive Singular Value Reconstruction

In view of the problem that the existing algorithms cannot extract the number of the reconstructed singular value accurately under low SNRs, this paper proposes a method based on the singular value difference spectrum to obtain the number of r adaptively. The singular value difference spectrum is defined as follows

(10) $$D_{i}=\delta_{i}-\delta_{i+1}$$

where i=1 , 2 , ⋯ , M−1 and $D=\left(D_{1},\;D_{2},\;\cdots ,\;D_{M-1}\right)$ is the singular value difference spectrum.

**Figure 3:**
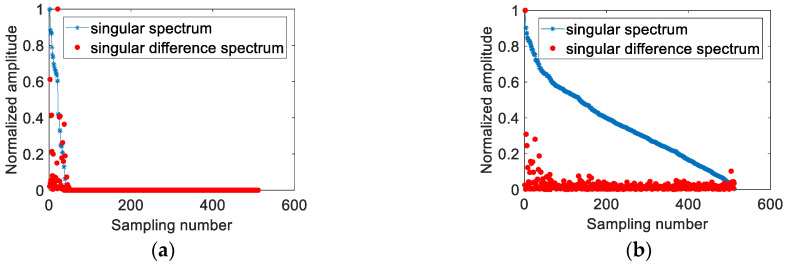
Singular spectrums and singular value difference spectrums, (**a**) the modulated signal without noise, (**b**) the white Gaussian noise.

As demonstrated in Figure 3a, the singular values in the first part of the modulated signal are large, and the latter part values are zero. According to (9), the original signal can be reconstructed by the nonzero singular values in the first part. Figure 3b shows that the difference spectrum of white Gaussian noise fluctuates significantly at the first few values and the later values are very small which tends to be zero. Under low SNRs, the noise proportion will increase in singular values, but the difference between the two adjacent singular values remains unchanged. Hence, the overall upward trend of the singular values can be eliminated in singular value difference spectrums.

The ASVR denoising method is realized in five steps.

Step1. Filter out small difference values; the threshold $D_{Th}$ is set as 0.03.

(11) $$D_{i}=\left\{ \begin{array}{ll} D_{i} & D_{i}\geq D_{Th}\\ 0 & D_{i}<D_{Th} \end{array}\right.$$

Step2. Define mutation sites (If there are multiple consecutive zero-value after a certain difference point, this point is defined as the mutation position).

Step3. Determine the number r of the useful singular values based on the demarcation point (Select the third mutation position as the demarcation point to prevent the interference caused by the nearness of the adjacent singular values of the modulated signals); the useless singular values are set to zero.

(12) $$\delta r_{i}=\left\{ \begin{array}{ll} \delta_{i} & i\leq r\\ 0 & i>r \end{array}\right.$$

Step4. Reconstruct the approximation matrix of δr.

(13) $$H_{R}=Udiag\left(\delta r_{i}\right)V^{T}$$

Step5. Restore the signal [27,28].

(14) $$Y_{R}\left(n\right)=\left\{ \begin{array}{ll} 1/n\sum_{j=1}^{n}H_{R}(j,n-j+1) & 1\leq n<K\\ 1/K\sum_{j=1}^{L}H_{R}(j,n-j+1) & K\leq n\leq M\\ 1/\left(N-n+1\right)\sum_{j=n-M+1}^{N-M+1}H_{R}(j,n-j+1) & M<n\leq N \end{array}\right.$$

**Figure 4:**
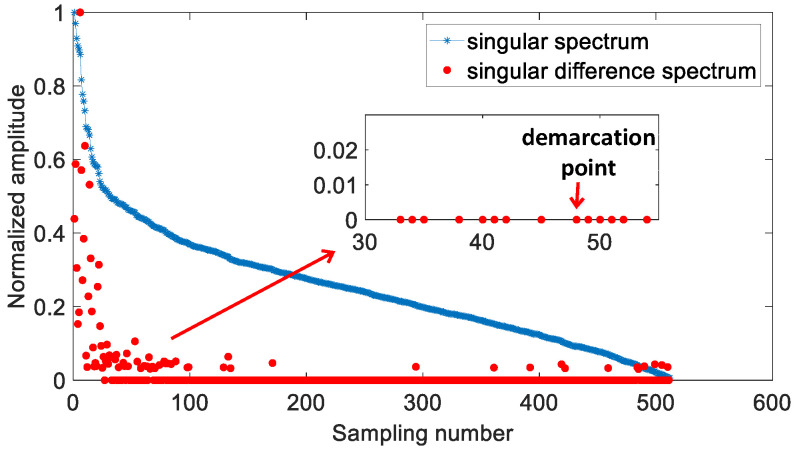
Singular spectrum and singular value difference spectrum of the received signal at the SNR of −6 dB.

The adaptive selection of effective singular values is realized in Step 1–3. Figure 4 shows the singular value difference spectrum after threshold filtering and the selection of a demarcation position based on the above principle.

**Figure 5:**
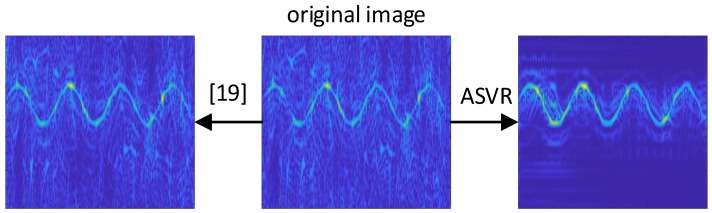
TFDIs of the raw signal at −6 dB and reconstructed signals based on ASVR and the method in [19].

In order to show the advantages of the ASVR algorithm, the comparison with the singular value average method in [19] is also given. Figure 5 shows the denoising effects of the singular value average method and the proposed method. When the SNR is −6 dB, compared with the raw signal, the reconstructed result of the existing approach is abysmal, which almost has no evident progress. However, the quality of the TFDI based on ASVR makes a significant improvement. Most noise is filtered out by ASVR, making the signal features more prominent. Simulation results prove the superiority of the ASVR algorithm under low SNRs.

### 3.2. TFDI Denoising Processing

After reconstructing the received signal using the ASVR algorithm, some noise still exists in the TFDI under low SNRs. Therefore, to further remove the noise and reduce the computational complexity, digital image processing methods are explored to obtain better features. In this part, the TFDI is processed into a binary image whose size is appropriate with four steps.

Step1. Normalize the original values of the SPWVD Time-Frequency spectrum and then form the grayscale TFDI.

Step2. The gray image is converted to a binary image based on the Otsu method [29].

(15) $$\underset{th}{\max}f\left(Th\right)={\left[\frac{1}{L_{1}}\sum_{n=1}^{L_{1}}G_{1}\left(n\right)-\frac{1}{L_{2}}\sum_{m=1}^{L_{2}}G_{2}\left(m\right)\right]}^{2}$$

(16) $$\begin{array}{ccc} if & A\left(i,j\right)\geq Th,\; & G_{1}=G(i,j)\\ & otherwise & G_{2}=G\left(i,j\right)\; \end{array}$$

where Th is the threshold, $L_{1}$ and $L_{2}$ are the length of $G_{1}$ and $G_{2}$, respectively. G(i,j) is the grayscale TFDI. 

The optimal threshold Th can be obtained by using the ergodic method to solve Equations (15) and (16). The binary image B can be expressed as

(17) $$B\left(i,j\right)=\left\{ \begin{array}{l} 1\;G\left(i,j\right)\geq Th\\ 0\;G\left(i,j\right)<Th \end{array}\right.$$

Step3. Considering that the binary images still have some isolated noise caused by the noisy environment and some processing noise generated in the kernel of SPWVD itself, morphologic filtering is applied to eliminate the noise further. Literature [30] indicates that a mathematic morphological algorithm is a powerful tool for image processing. The algorithm processes images using morphological transforms according to the local shape features of images by appropriate structure elements [31]. Thus, the main shape features of images are preserved while filtering small background noise. Dilation and erosion are fundamental morphological operations. Dilation can make the highlighted area of the image grow gradually by calculating the maximum value of the pixel in the area covered by the structure element and assigning the utmost value to this pixel. The dilation of an image I by a structure element S, denoted as I⊕S [32], can be written as

(18) $$\left(I⊕S\right)\left(\vec{p}\right)=\underset{x\in E}{\max}\left\{I\left(x\right)+S\left(\vec{p}-x\right)\right\}$$

where $\vec{p}$ is the pixel position in the image, x∈E represents all points in the image.

In contrast, the purpose of the erosion operation is to assign the minimum value of the pixel in the area covered by the structure element to the corresponding pixel. The edge of the highlighted area of the image can shrink inward, and small meaningless objects in the image can be eliminated. The erosion of an image I by a structure element S, denoted as IΘS, is expressed as

(19) $$\left(I\Theta S\right)\left(\vec{p}\right)=\underset{x\in E}{\min}\left\{I\left(x\right)+S\left(\vec{p}-x\right)\right\}$$

This paper uses structure elements with a radius of four pixels to filter the image. First, eliminate small objects through the cascade of Θ and ⊕. Then, fill the small cavities in the object and smooth the boundary through the cascade of ⊕ and Θ. The structure of the morphologic filter designed in this paper is as follows

(20) M=(((BΘR)⊕R)⊕R)ΘR

where B is the binary image obtained by previous steps, R is the structure element, M is the filtered image. Then, to remove the isolated noises, the pixel number of each connected group is calculated. Furthermore, the group of pixels whose size is smaller than the 10% of the largest group is removed (pixel value is set to zero).

Step4. Bicubic interpolation is applied to adjust image size [33]. The output pixel value can be expressed as

(21) $$p\left(x,y\right)=\sum_{i=0}^{3}\sum_{j=0}^{3}f\left(x_{i},y_{i}\right)W\left(x-x_{i}\right)W\left(y-y_{i}\right)$$

(22) $$W\left(x\right)=\left\{ \begin{array}{ll} 1.5{\left|x\right|}^{3}-2.5{\left|x\right|}^{2}+1 & for\;\left|x\right|\leq 1\\ -0.5{\left|x\right|}^{3}+2.5{\left|x\right|}^{2}-4\left|x\right|+2 & for\;1<\left|x\right|<2\\ 0 & otherwise \end{array}\right.$$

where W(x) is the interpolation basis function. p(x,y) is a weighted average of pixels in the nearest 4-by-4 neighborhood, which allows it to create smoother image edges than bilinear interpolation [34].

**Figure 6:**
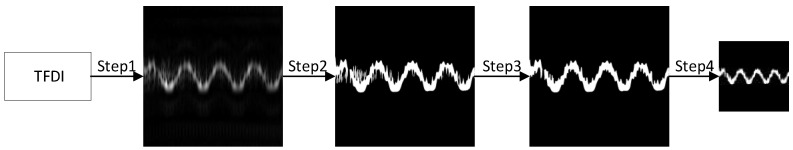
The processes of the TFDI denoising (SFM is selected at −6 dB as an example).

The processes of the TFDI denoising are shown in Figure 6. Though the above processing, even at −6 dB, the TFDI of the signal is still evident, ensuring the accuracy of recognition.

## 4. Classification

### 4.1. Deep Residual Learning

DCNN is a kind of feedforward Neural Network, which has an excellent performance in image processing [35]. It can directly utilize original images as the input. This avoids the complicated preprocessing of the image. Thus, DCNN has been widely used in image classification, target positioning and other fields. 

DCNN integrates multilevel features [36] and classifiers, and the “levels” of features can be enriched by stacking layers. Studies have indicated that network depth is critical to feature extraction accuracy [36]. Hence, classic networks [37,38] on the challenging ImageNet dataset [39] all adapt deep models. 

**Figure 7:**
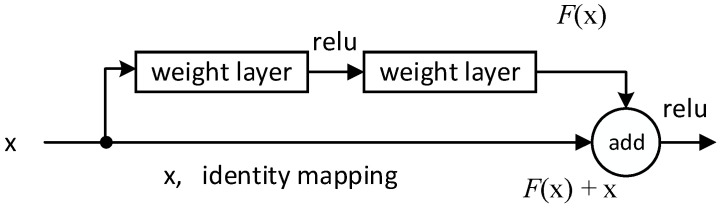
The structure of a residual block.

In general, the performance of the network will be enhanced with increased depth. However, naively adding the layers result in the accuracy getting saturated and then degrades rapidly [40]. The vanishing gradient and the problem of network degradation will both arise when the network is too deep. As is demonstrated in Figure 7, residual networks [41] add an “expressway (shortcut connections)” to skip one or more layers to persist the significant weights and parameters from earlier layers throughout the end. Thus, problems in depth network are solved.

The output of the residual block can be written as

(23) $$y=\sigma (x+F(W_{2}\sigma (W_{1}x)))$$

where x and y are the input and output of the layers. The function $F(x,W_{i})$ is residual mapping. σ is the activation function ‘‘relu’’.

Assume that the activation function is a direct mapping, $x_{l}$ and $x_{l+1}$ are the input and output of the layers; for a deeper layer L, the relationship with the layer l can be expressed as

(24) $$x_{L}=x_{l}+\sum_{i=l}^{L-1}F(x_{i},W_{i})$$

The output of L can be represented by the $x_{l}$ and the sum of residual parts of the middle layers. The gradient of the loss function ε with respect to $x_{l}$ can be expressed as

(25) $$\frac{\partial \varepsilon }{\partial x_{l}}=\frac{\partial \varepsilon }{\partial x_{L}}\frac{\partial x_{L}}{\partial x_{l}}=\frac{\partial \varepsilon }{\partial x_{L}}\left(1+\frac{\partial }{\partial x_{l}}\sum_{i=l}^{L-1}F(x_{i},W_{i})\right)=\frac{\partial \varepsilon }{\partial x_{L}}+\frac{\partial \varepsilon }{\partial x_{L}}\frac{\partial }{\partial x_{l}}\sum_{i=l}^{L-1}F(x_{i},W_{i})$$

$\frac{\partial }{\partial x_{l}}\sum_{i=l}^{L-1}F(x_{i},W_{i})$ cannot be negative ones throughout the training process. Thus, the problem of vanishing gradient will not occur in the residual network. Besides, the gradient $\frac{\partial \varepsilon }{\partial x_{L}}$ of the layer L can be directly passed to any layer l shallower than it so that network degradation will no longer exist.

### 4.2. Network Architecture

ResNet-50 [41], one of the classic deep residual networks, has shown extremely high classification accuracy and good generalization abilities on the database of ImageNet Large-Scale Visual Recognition Challenge (ILSVRC). This paper designed a deep residual network based on the structure of ResNet-50. The input size of the network is adjusted to 1 × 64 × 64 to reduce the amount of calculation and adapt to the single-channel TSDIs data set. The structure of the network is described in Table 1. First, the network performs a convolution operation on the input, and then contains four residual blocks to extract image features. A total of 50 weight layers are included in the network. The training parameters of each weight layer are transmitted through ‘‘shortcut connections’’. Thus, the vanishing gradient and network degradation will no longer trouble the network. Rich convolutional layers can extract features as accurately as possible.

**Figure 8:**
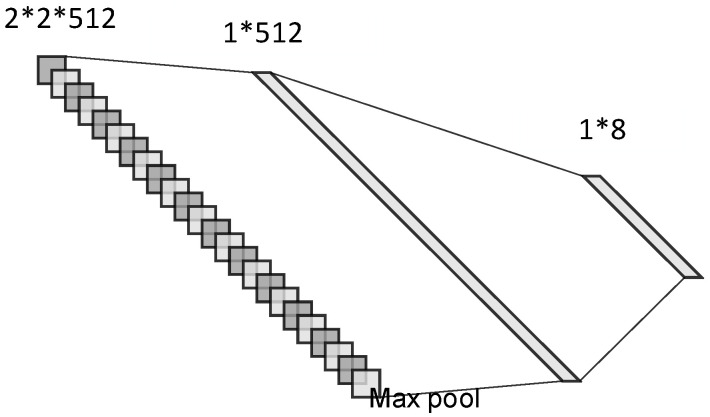
The structure diagram of dimensionality reduction and classification layers.

In order to reduce the dimensionality of the network output features, a global maximum pooling layer is added at the end of the network, which avoids the ambiguity caused by the average pooling operation. The reduced-dimensional features are sent to the multilayer perceptron to train the classification network. The structure diagram of the global maximum pooling layer and classification layers is demonstrated in Figure 8. Then, the problem of modulation recognition is transformed into the issue of image classification. A part of the TFDIs is selected randomly as learning data to complete the training process of the network. Using the newly trained network to test the rest TFDIs, accurate recognition of various radar signals can be realized.

## 5. Simulation Result

In this section, the newly trained network is applied to evaluate the robustness of the proposed approach. Eight types of simulation radar signals are LFM, SFM, EQFM, FSK, 4FSK, BPSK, Frank code and CW signals. Gaussian white noise is added to the signals. Table 2 shows the parameters of the signals. 

For each signal, we take 200 samples every 2 dB when the SNR ranges from −12 dB to 2 dB. 50% of samples are selected randomly to achieve the training process of the residual network and the rest are chosen as the testing set. There are 6400 training samples and 6400 test samples in total. For each classification, we carry out five experiments and calculate the average value of the five experiments as the final accuracy. All experiments are carried out in MATLAB R2017b which is supported by a computer with Intel i5 9700 CPU and NVIDIA GeForce GTX 1060 3 GB GPU hardware capabilities.

### 5.1. Recognition Result

The SNR and the probability of successful recognition (PSR) are two important parameters to describe the recognition results. The SNR is defined as $SNR=10\log_{10}\left(\sigma_{s}^{2}/\sigma_{n}^{2}\right)$, where $\sigma_{s}^{2}$ and $\sigma_{n}^{2}$ are the signal variance and the noise variance, respectively. The function of the PSR is as $PSR=\left(\sum_{i=1}^{N}TP_{i}\right)/\left(\sum_{i=1}^{N}\left(TP_{i}+FP_{i}\right)\right)$, where $TP_{i}$ is the number of samples i that were correctly identified, $FP_{i}$ is the number of samples i that were incorrectly identified. For example, the PSR under −4 dB means the ratio of the correctly identified samples under −4 dB by the system to all samples under −4 dB. The PSR for FSK under −4 dB means the rate of the correctly identified FSK samples under −4 dB by the system to all FSK samples under −4 dB.

**Figure 9:**
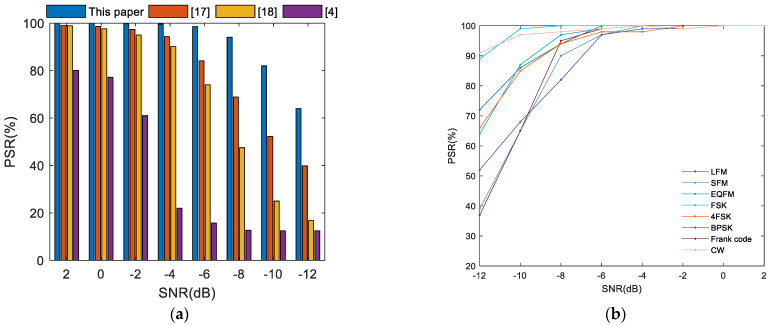
Recognition performance as a function of the SNR. (**a**) Overall recognition accuracy; (**b**) Recognition accuracy of eight kinds of signals.

The recognition results of the eight radar signals under different SNRs are vividly shown in Figure 9. It can be seen that there is a positive correlation between the PSR and the SNR. When the SNR exceeds −4 dB, the overall PSR of the method is nearly 100%. When the SNR is below −4 dB, the overall PSR enhances significantly with the increase of the SNR. Even when the SNR is −8 dB, the overall accuracy still exceeds 94%. The system also maintains a PSR of more than 80% for FSK, 4FSK, LFM, EQFM and CW signals at the SNR of −10 dB. The PSR of the system will be unsatisfactory when the SNR ≤ −12 dB. This proves that the proposed method is effective and robust. 

Figure 9a illustrates that the recognition result of the proposed method precedes the current methods [4,17,18] comprehensively under the same SNR. When the SNR is −8 dB, the recognition result of the existing approach is very poor. As a contrast, the overall PSR of the proposed method can exceed 94% in the same condition. This proves that the proposed method has better antinoise performance and higher recognition precision than the previous approach. 

This is mainly because this paper improves the methods of signal preprocessing and TFDIs processing. Firstly, ASVR is proposed for the first time and utilized to repair signals before extracting TFDIs. This removes the majority of the noise in received signals. Secondly, the system performs morphologic filter processing on binary TFDIs, which further reduces noise and improves the quality of TFDIs. The measures mentioned above ensure that the system has a more robust antinoise performance than the previous method. Thirdly, the newly designed network has rich convolutional layers and a residual structure. Thus, the network has a powerful feature extraction capability without reducing the accuracy due to degradation and gradient disappearance. Overall, these three points make more accurate classification decisions of the system, and the simulation analysis will further prove it later.

**Figure 10:**
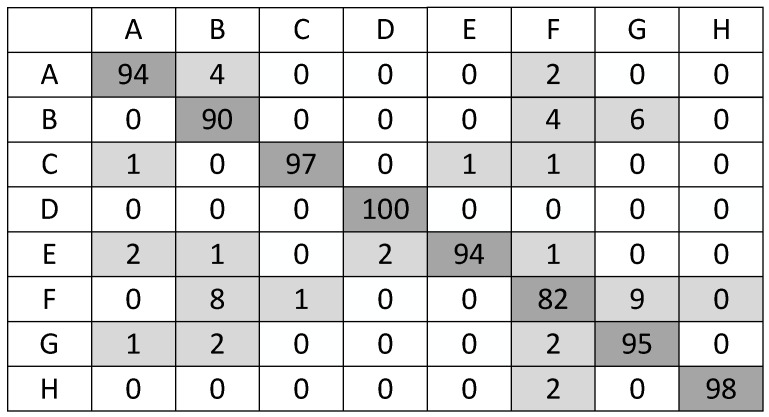
Confusion matrix for the system at the SNR of −8 dB.

Figure 10 shows the details of the classification at the SNR of −8 dB. It is manifested from the confusion matrix that the system still has a satisfactory recognition performance. The recognition mistakes mainly occur between the signal pairs with similar TFDIs, such as BPSK and Frank code signals, SFM and Frank code signals, SFM and BPSK signals. The submergence of some frequency information in intense noise causes ASVR to be unable to reconstruct all useful frequency components in the original signal. The process of image resizing also makes small frequency jump blurred. The losses and blurs of this small frequency information finally lead to confusion between signals.

### 5.2. Effects of ASVR

**Figure 11:**
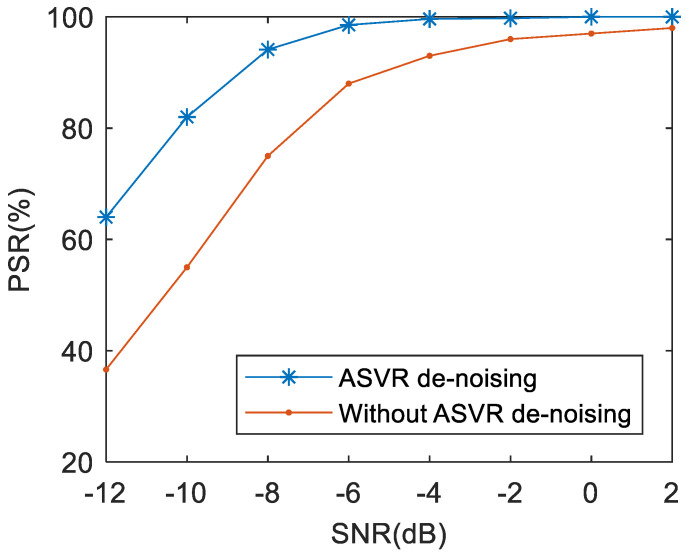
The effects of the ASVR denoising method on the PSR of the proposed approach.

In order to analyze the effects of the ASVR denoising algorithm, experiments based on the system without ASVR are also carried out. Figure 11 indicates that the recognition results of the system with the ASVR denoising preprocessing are significantly better than those without ASVR preprocessing. This is mainly because ASVR filters most of the noise out of the frequency band, which repairs the original signals. Hence, recognition results are more precise.

### 5.3. Effects of Morphologic Filtering

**Figure 12:**
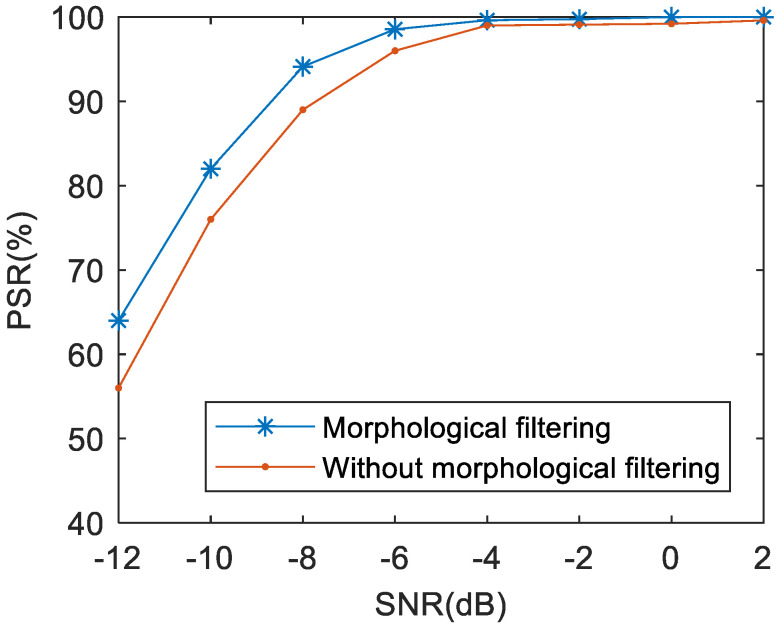
The effects of the morphologic filtering on the PSR of the proposed approach.

In this part, the new learned network is used to evaluate the validity of the morphologic filtering. Figure 12 shows the effects of morphologic filtering through comparative experiments. According to the picture, the recognition result of the system without morphologic filtering is obviously lower than that of the system with morphologic filtering in the same SNR condition. This proves that morphologic filtering can effectively improve the quality of TFDIs. This is due to the fact that the application of morphologic filtering can further remove the noise in the frequency band and smooth the edge of the image.

### 5.4. Effects of Networks

**Figure 13:**
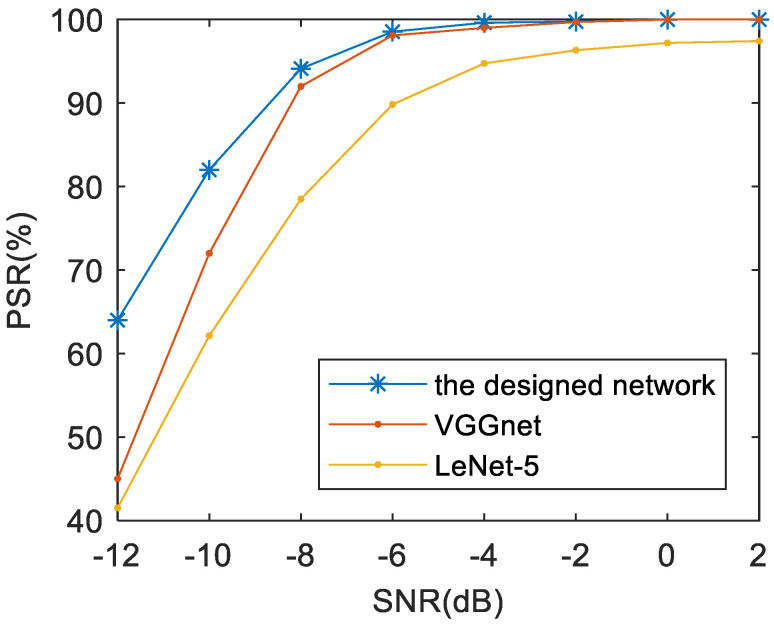
The effects of different networks on the PSR of the proposed approach.

In addition, experiments based on several classic networks [36,42] are designed to explore the effects of different types of DCNNs. The training TFDIs are adjusted to the same size as each network input, respectively (VGGnet with the input size of 224 × 224 pixels, LeNet-5 with the input size of 28 × 28 pixels). The comparison results are shown in Figure 13. It can be clearly seen that the depth networks have a better performance on final recognition accuracy than shallow CNN Lenet-5. Although deep structures are used, the network designed in this paper is also superior to VGGnet due to its residual design. Besides, using a smaller convolution kernel instead of a large convolution kernel, the training and testing time of the newly designed network is much shorter than that of VGGnet. Hence, it is reasonable to select the designed network as the classifier in this paper.

## 6. Conclusions

Accurate identification of the modulation type of the radar signals plays a prominent part in modern electronic countermeasures. In this paper, the ASVR algorithm with good antinoise capability and strong adaptability is proposed for the first time. Then, deep residual learning is combined with ASVR to identify various radar signals. This method can identify eight types of radar signals (including LFM, SFM, EQFM, FSK, 4FSK, BPSK, Frank code and CW signals) effectively under low SNRs. Simulation results show that the overall PSR of eight radar signals can still reach 94.1% even when the SNR is −8 dB. This confirms the superiority and robustness of the proposed method. The research has great significance in electronic reconnaissance, electronic protection and other fields of modern electronic countermeasure. However, the study is only focused on single-modulation radar signals. How to realize the recognition of multimodulation radar signals has become a problem to be solved. This is a crucial point in our future research work.

## Figures and Tables

**Table 1:** Architecture of the deep residual network designed in this paper.

Layer	Conv1	Conv2_x		Conv3_x	Conv4_x	Conv5_x
Output size	32 × 32	16 × 16		8 × 8	4 × 4	2 × 2
network	7 × 7, 64, stride 2	3 × 3, Max pool, stride 2	$\left[\begin{array}{l} \;1\times 1,\;16\\ \;3\times 3,\;16\\ \;1\times 1,\;64 \end{array}\right]\times 3$	$\left[\begin{array}{l} \;1\times 1,\;32\\ \;3\times 3,\;32\\ \;1\times 1,\;128 \end{array}\right]\times 4$	$\left[\begin{array}{l} \;1\times 1,\;64\\ \;3\times 3,\;64\\ \;1\times 1,\;256 \end{array}\right]\times 6$	$\left[\begin{array}{l} \;1\times 1,\;128\\ \;3\times 3,\;128\\ \;1\times 1,\;512 \end{array}\right]\times 3$

**Table 2:** Parameters of simulation radar signals.

Signal	Parameter	Ranges
LFM	$\mathrm{Carry}\;\mathrm{frequency}\left(f_{c}\right)$	U(0.1~0.4)
	Bandwidth(Δf)	U(0.1~0.4)
SFM	$f_{c}$	U(0.1~0.4)
	Δf	U(0.1~0.4)
EQFM	$f_{c}$	U(0.1~0.4)
	Δf	U(0.1~0.4)
FSK	$f_{c1}$,$f_{c2}$	U(0.1~0.4)
	$T_{s}$	N(1/32~1/8)
4FSK	$f_{c1}$,$f_{c2}$,$f_{c3}$,$f_{c4}$	U(0.1~0.4)
	$T_{s}$	N(1/32~1/8)
BPSK	$f_{c}$	U(0.1~0.4)
	Barker codes	[5,7,11,13]
	$T_{s}$	N(1/64~32)
Frank code	$f_{c}$	U(0.1~0.4)
	$T_{s}$	N(1/100~1/64)
	Phase number	[4,5,6]
CW	$f_{c}$	U(0.1~0.4)

Where U(⋅) and N(⋅) means a uniform distribution based on the sampling frequency and the length of the signal, respectively. For example, if the carrier frequency $f_{c}=3\mathrm{GHz}$ and the sampling frequency $f_{s}=15\mathrm{GHz}$ are set, the uniform result can be defined as $f_{c}=U(f_{c}/f_{s})=U(1/5)$. U(0.1~0.4) denotes that the frequency is a uniform distribution from $0.1*f_{s}$ to $0.4*f_{s}$. N(1/32~1/8) means the distribution of points is uniform and the range is between N/32 and N/8.

## Data Availability

No new data were created or analyzed in this study. Data sharing is not applicable to this article.

## Definition

This article uses various expressions when describing the accuracy of the recognition system, including the probability of successful recognition, the recognition rate, the accuracy and the PSR. The equations for these descriptions are the same as Equation (11).
